# Association Between Late-Onset Leukoencephalopathy With Vanishing White Matter and Compound Heterozygous EIF2B5 Gene Mutations: A Case Report and Review of the Literature

**DOI:** 10.3389/fneur.2022.813032

**Published:** 2022-06-16

**Authors:** Fanxin Kong, Haotao Zheng, Xuan Liu, Songjun Lin, Jianjun Wang, Zhouke Guo

**Affiliations:** ^1^Encephalopathy and Psychology Department, Shenzhen Traditional Chinese Medicine Hospital, Shenzhen, China; ^2^The Fourth Clinical Medical College of Guangzhou University of Chinese Medicine, Shenzhen, China; ^3^Shenzhen Traditional Chinese Medicine Hospital, Shenzhen, China

**Keywords:** leukoencephalopathy with vanishing white matter, ovarian failure, adult-onset, EIF2B5, compound mutation

## Abstract

Leukoencephalopathy with vanishing white matter (LVWM) is an autosomal recessive disease. Ovarioleukodystrophy is defined as LVWM in females showing signs or symptoms of gradual ovarian failure. We present a 38-year-old female with ovarioleukodystrophy who showed status epilepticus, gait instability, slurred speech, abdominal tendon hyperreflexia, and ovarian failure. Abnormal EEG, characteristic magnetic resonance, and unreported EIF2B5 compound heterozygous mutations [c.1016G>A (p.R339Q) and c.1157G>A (p.G386D)] were found. Furthermore, the present report summarizes 20 female patients with adult-onset ovarioleukodystrophy and EIF2B5 gene mutations. In conclusion, a new genetic locus for LVWM was discovered. Compared with previous cases, mutations at different EIF2B5 sites might have different clinical manifestations and obvious clinical heterogeneity.

## Introduction

Leukoencephalopathy with vanishing white matter (LVWM; MIM# 603896) is a progressive central nervous system condition that can be divided into the congenital, infant acute, early childhood-onset, juvenile-onset, and late-onset types (1, 2). The symptoms include ataxia, spasticity, and optic atrophy (3–5). Most patients display symptoms in childhood, but the symptoms can appear or progress rapidly after trauma or stress (3–5). Unfortunately, there is no curative treatment for LVWM (3–5).

Mutations in the *EIF2B1, EIF2B2, EIF2B3, EIF2B4*, and *EIF2B5* genes, which encode the subunits of the eIF2B protein, have been associated with LVWM (6). eIF2B participates in the normal regulation of protein synthesis in cells. A mutation in any of the five genes can cause LVWM in an autosomal recessive manner (3–5). Cells with mutations in eIF2B have dysregulated protein synthesis and are susceptible to changes in environmental conditions and stress (3–5).

Some female patients can also suffer from ovarian hypoplasia, and LVWM might be related to decreased levels of sexual hormones before menopause, manifesting as primary amenorrhea or secondary amenorrhea, lasting for more than 6 months. At least three cases of co-occurrence of leukodystrophy and premature ovarian failure have been reported (7–9). Ovarioleukodystrophy is the proper term that describes this condition (10). This case report presents a case of sudden progression of adult-onset ovarioleukodystrophy associated with compound heterozygous *EIF2B5* mutations. Furthermore, the up-to-date clinical features about late-onset ovarioleukodystrophy associated with *EIF2B5* gene mutations were reviewed.

## Case Report

A 38-year-old Chinese female, the second child of a non-consanguineous marriage, presented a 4-year history of unsteady walking and a 10-day history of slurred speech. She was healthy until the onset of these symptoms. About 4 years ago, the patient developed an unsteady gait when walking and occasional falls, without obvious incentives. She gradually developed procrastination, poor memory, occasional incontinence, slow speech, slow thinking, and menometrorrhagia, but she could still care for herself. Family members reported that the patient's menstrual cycle was irregular and that her menstrual flow had decreased in recent years. Because the symptoms were gradually aggravating, her siblings took her to the local hospital. Ten days before hospitalization, the patient began to have slurred speech, abdominal bloating, and constipation and became unable to walk without help. She had no fever, chills, headache, nausea, vomiting, abdominal pain, or diarrhea. She did not get better after treatment at a local clinic, but no treatment information could be obtained.

She was admitted to our hospital emergency department. She was unconscious and was transferred to the encephalopathy department. A little wet rale was heard in both lower lungs on auscultation. Neurological examination showed that the bilateral radial periosteal reflex, biceps, and triceps tendons were hyper reflexive, but the bilateral knee reflex and Achilles tendon reflex were weak. Abdominal wall reflex was not elicited. Bilateral pathological signs were negative. There was no resistance in the neck, and meningeal irritation was negative. The other neuro-examinations could not be performed. The patient had no fever, but the blood tests indicated infection: white blood cells (WBC) at 9.65 (reference: 3.50–9.50) × 10^9^/L, neutrophil % at 80.3% (reference: 40.0–75.0%), and high- sensitivity C-reactive protein (hs-CRP) at 69 (reference: 0–10) mg/L, erythrocyte sedimentation rate (ESR) at 106 (reference: 0–20) mm/h. Serum sex hormones showed ovarian failure, with estradiol at 55 pmol/L (reference: 56–1,625) IU/L. The other endocrine hormones in serum were abnormal either, such as total triiodothyronine (tT3) at 0.86 (reference: 1.01–2.48) nmol/L, prolactinemia at 835 (reference: 58–412) mIU/L. The other tests showed homocysteinemia at 22.5 (reference: 0–15.0) μmol/L and antinuclear antibody (1:100) (±). HIV antibody, HBV antibody, HCV antibody, glucose, lipids, glycosylated hemoglobin, tumor carbohydrate antigen, AMA profile, ANA profile, ANCA, anti-keratin antibody, anti-myeloperoxidase antibody, and anti-cyclic citrulline peptide antibody were all negative.

Computed tomography (CT) scan confirmed the infection diagnosis of peritonitis, pneumonia and cholecystitis which were associated with her abdominal bloating and constipation. Much more abnormality was found on CT, such as multiple ground-glass nodules in the upper lobe of the right lung, dilated kidneys and entire bilateral ureters. Bilateral mild hydrops, dilated upper right kidney ureter, and minor ascites around the appendix were found by further ultrasound.

**Figure 1:**
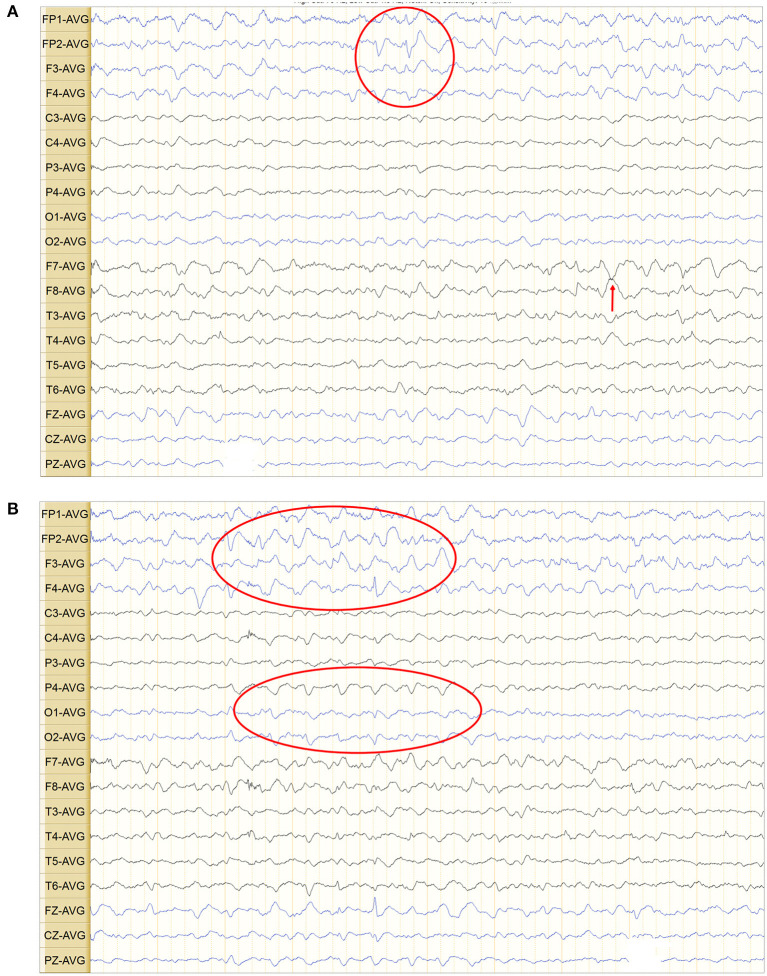
**(A,B)** Multiple pathological waves, such as spikes, sharp, spike-slow, and mixed spike-slow waves, can be seen on electroencephalogram in the bilateral frontotemporal areas, bilateral temporal areas, bilateral midfrontal areas, and bilateral occipital areas (red ovals and red circle). Sharp waves (red arrows) were also visible in the central-parietal area.

**Figure 2:**
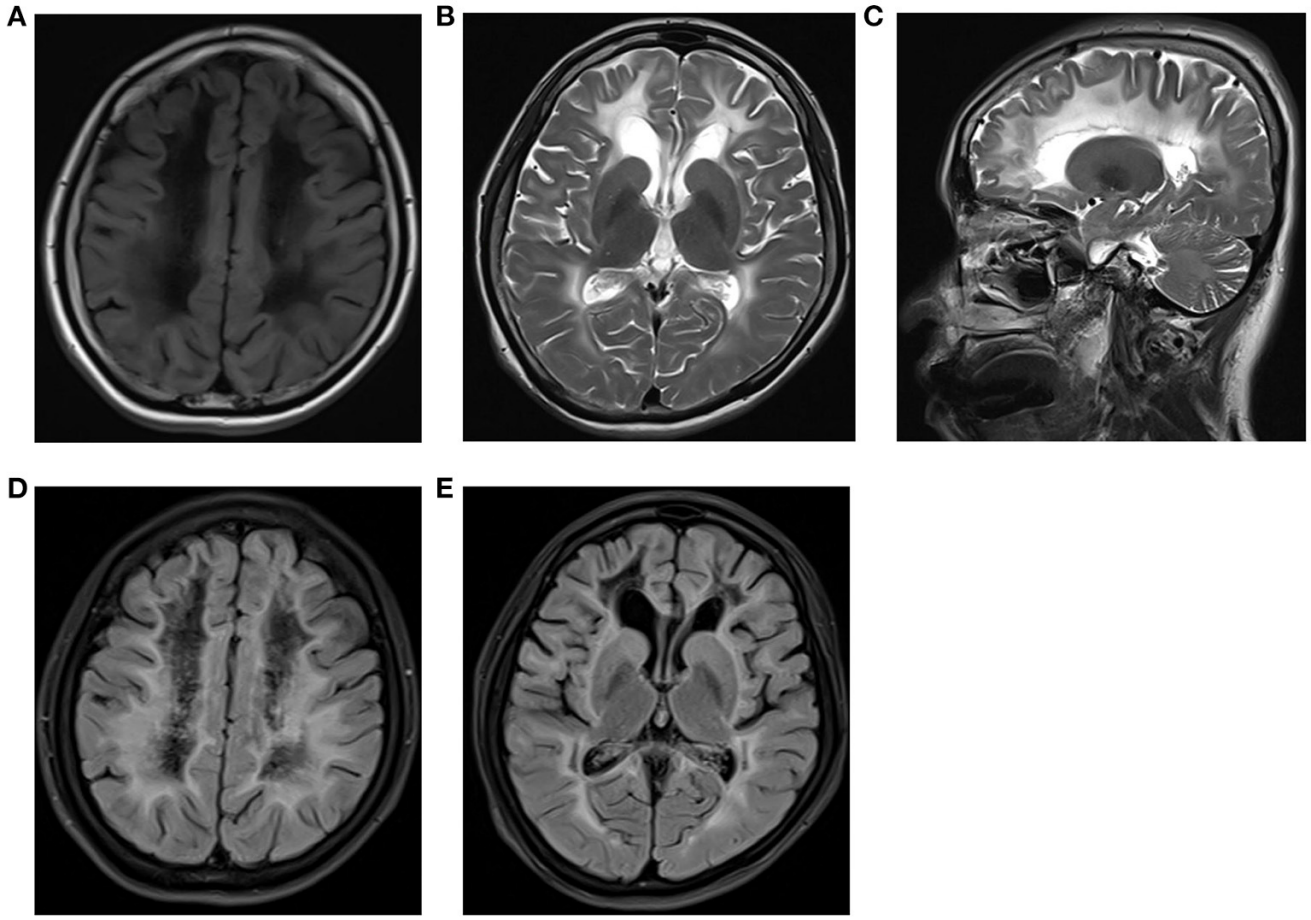
Magnetic resonance imaging showing extensive and symmetrical changes in bilateral cerebral hemisphere demyelination with glial hyperplasia involving the corpus callosum. Obvious brain atrophy was also observed. **(A)** Axial T1-weighted. **(B)** T2- weighted. **(C)** Coronal T2-weighted. **(D, E)** T2Flair.

The cerebrospinal fluid pressure was 82 mm H_2_O and showed WBC at 35 × 106/L, glucose at 5.18 mmol/L, chlorine at 113.0 mmol/L, and immunoglobulin G at 39.8 mg/L suggesting subspinous intracranial infection associated with her slurred speech. Cerebrospinal fluid autoimmune encephalitis antibody spectrum, Bacteria (aerobic and anaerobic), fungal culture, and second-generation gene sequencing of pathogenic microorganisms were negative. Cytological pathology was negative. The electroencephalogram was abnormal. Multiple pathological waves, spikes, sharp, spike-slow, and mixed spike- slow waves were found in bilateral frontotemporal and midfrontal areas. Sharp waves were also seen in the bilateral temporal, central-parietal, and occipital areas (red marks on Figure 1). Brain magnetic resonance imaging (MRI) showed extensive and symmetrical changes in bilateral cerebral hemisphere demyelination with glial hyperplasia involving the corpus callosum (Figure 2). Magnetic resonance angiography (MRA) was normal.

On December 8, 2020 the patient began to have partial epilepsies and developed status epilepticus. Intravenous midazolam and intramuscular phenobarbital had to be used. The combination of valproate, oxcarbazepine, and levetiracetam could reluctantly control epilepsy. The patient never regained consciousness. Due to high fever, pulmonary failure secondary to aspiration pneumonia, and uncontrollable status epilepticus, the patient was transferred to the ICU and then for hospice care. The patient died on January 1, 2021.

## Gene Search

The clinical characteristics of the patient, as well as the MRI presentation of cerebral white matter, guided the path of possible causative gene testing. Genomic DNA was extracted from the patient's blood. The Illumina NextSeq CN500 sequencing platform was used to sequence the gene capture. The Agilent SureSelect Human All Exon V6 capture kit was used to capture and enrich the entire exome region as well as some flanking regions. The research focused on 145 genes linked to hereditary cerebral leukodystrophy and those linked to the patient's clinical phenotype. BWA-men was used as the sequence aligner on the Sequence Platform, and GATK software was used as the variant caller. The reference sequence for the positive gene locus is NG 015826.1(https://www.ncbi.nlm.nih.gov/nuccore/NG_015826.1?from=5337&to=15290&report=genbank). Variants that were clearly or potentially associated with the subjects' clinical phenotype were screened. Finally, Sanger sequencing was used to validate the results. Two mutations were located on chromosome 3 at chr3:183858378 and chr3:183859713. Sequencing revealed that the patient had one heterozygous mutation of the *EIF2B5* gene in exon 7, changing the arginine into glutamine residue at position 339 (chr3:183858378, NM_003907.3: c.1016G>A). The frequency of this variant in the common people database was ExAC: 0.000099, gnomAD: 0.000060, and it was classified as pathogenic/possibly pathogenic by the ClinVar database. At the same amino acid position, two other mutations were reported as pathogenic variants (c.1016G>C, c.1015C>T) (11, 12). Another heterozygous mutation was found in exon 8, changing the glycine into aspartic acid at position 386 (chr3:183859713, NM_003907.3: c.1157G>A). This variant was located in the near splice-site region. The ADA score of the dbscSNV, a splice site variant hazard prediction software, was >0.6, indicating that it influenced splicing. The variant at the same locus of the EIF2B5 gene (NM_003907.3: c.1157G>T, G386V) was reported as pathogenic mutation (11). NM 003907.3: c.1016G>A p.R339Q variations were pathogenic/probably damaging, according to bioinformatics prediction software (PolyPhen-2, Mutation Taster, REVEL) (0.822, 1.000, 0.789). The prediction scores for NM 003907.3: c.1157G>A p.G386D were 1.000, 1.000, and 0.985, indicating pathogenic or detrimental potential. Therefore, we considered these two new variants to be likely compound pathogenic/harmful heterozygous EIF2B5 gene mutations. Based on the patient's clinical features, MRI presentation, and the above-mentioned predictable results. None of the other detected genetic variants were consistent with the patient's characteristics. Other EIF2B-related gene variants or LVWM-causing genes, such as AARS2, were not discovered at the same time.

## Discussion

LVWM is a disease related to white matter cystic degeneration and tissue loss (3). A cavitating leukoencephalopathy is observed (2), with a radial cobweb-like pattern of remaining fiber bundles in the brain lobe, U fibers relatively preserved and myelinated, rare myelin, and cystic degeneration coexisting with oligodendrocytosis (3).

The essential function of EIF2B is reflected by the evolutionary conservation of the complex in eukaryotes (13). EIF2B is a nucleotide-exchange factor and can continuously catalyze the recycling of eIF2 in peptide chain translation, turning inactive eIF2-GDP into active eIF2-GTP. This cycle is the key point to regulate translation, and it is regulated by many physiological and pathological conditions (11, 14).

We list the clinical features and variant genes of female late-onset ovarioleukodystrophy due to the EIF2B5 gene mutation in Table 1. R113H was the most common mutation in a previous LVWM report (15). This mutation was found in 76.2% (16/21) of 21 cases; 57.3% (12/21) were homozygous, and 19.1% (4/21) were heterozygotes with c.338G>A (Arg113His) on exon 3. The other homozygous cases were c.545C>T (Thr182Met) on exon 4 (patient #9) and c.1759A>G (Ile587Val) on exon 13 (patient #20). The other homozygous cases were c.545C>T (Thr182Met) on exon 4 (patient #9) and c.1759A>G(Ile587Val) on exon 13 (patient #20). Patient #2 and the patient reported here had uncommon *EIF2B5* gene mutations. Almost all cases of ovarioleukodystrophy are due to exon mutations, except for patient #1. The intronic variant in the *EIF2B5* gene activates a cryptic 5' donor splice site of intron 7, probably leading to the synthesis of a truncated protein (16).

**Table 1:** Literature review for female late-onset ovarioleukodystrophy due to the EIF2B5 gene mutation.

**#**	**Onset age**	**Exon**	**Nucleotide change**	**Amino-acid change**	**Mood/Cognitive change^**a**^**	**Seizures^**b**^**	**Other clinical features^**c**^**	**Reference**
1	26	7	c.725A>G(A725G)	Tyr242Cys(Y242C)	(-)	(-)	Right hand clumsiness, dystonic left foot postures, generalized hyperreflexia	(16)
		Intronic	c.1156 + 13G>A					
2	19	7	c.896G>A (G896A)	Arg299His (R299H)	NM	GE, tonic-clonic seizure	Episodes of left-sided weakness, paranesthesia, migraine, postural upper limb tremor, spastic tetraparesis, saccadic interposition of smooth pursuit, visual hallucinations	(7)
		5	c.913A>T(A913T)	Met305Leu (M305L)				
3	19	3	c.338G>A (G338A)	Arg113His (R113H)	NM	Epileptic seizures	NM	(9)
		7	G896A	R299H				
4	31	3	G338A	R113H	anxiety, depression	SE	Spasticity, GI, headaches,	(20, 21)
			G338A	R113H				
5	48	3	G338A	R113H	CI, MMSE:21	simple seizures	Spasticity, GI	
			G338A	R113H				
6	16	3	G338A	R113H	NM	NM	NM	
		4	c.583C>T (C583T)	Arg195Cys (R195C)				
7	19.5	3	G338A	R113H	NM	NM	Motor signs (potentially coupled with acute episode)	
		NM	NM	NM				
8	27	3	G338A	R113H	NM	NM	Motor signs	
			G338A	R113H				
9	45	4	c.545C>T (C545T)	Thr182Met (T182M)	Euphoric, emotionally unstable, CI, disorganized, pathological theft, delusional behavior	NM	GI, exaggerated tendon reflexes	[38]
			C545T	T182M				
10	32	3	G338A	R113H	CI	NM	Horizontal nystagmus, CA, tetraplegia,	[39]
			G338A	R113H				
11	25	3	G338A	R113H	NM	NM	GI, speech difficulties, spasticity, CA	(15)
			G338A	R113H				
12	42.5	3	G338A	R113H	CI, MMSE:23	(-)	CA	(31)
			G338A	R113H				
13	33	3	G338A	R113H	Depression	GE	NM	
			G338A	R113H				
14	27	3	G338A	R113H	NM	GE, SE	NM	
			G338A	R113H				
15	21	3	G338A	R113H	CI, MMSE:25	GE	CA	
		7	G896A	R299H				
16	30	3	G338A	R113H	CI, MMSE:21	partial epilepsy	CA	
			G338A	R113H				
17	35	3	G338A	R113H	CI, MMSE:4	GE	CA, spastic paraplegia	
			G338A	R113H				
18	17	3	G338A	R113H	CI, MMSE:0	GE	Tetraplegia	
			G338A	R113H				
19	18	3	G338A	R113H	CI, MMSE:15	NM	CA, spastic paraplegia	
			G338A	R113H				
20	22	13	c.1759A>G(A1759G)	Ile587Val (I587V)	Attention impairment, delirium, taciturnity, hypomnesia, hypologia	NM	Urinary incontinence	(8)
			A1759G	I587V				
21	38	7	1016G>A	Arg339Gln(R339Q)	CI	partial epilepsy, SE	GI, slurred speech, abdominal bloating, upper limps tendon hyperreflexia, lower limps tendon hyporeflexia	Our patient
		8	1157G>A	Gly386Asp (G386D)				

*NM, Not mentioned*.

a *CI, Cognitive impairment; MMSE, Mini-mental State Examination*.

b *GE, generalized epilepsy; SE, Status epilepticus*.

c *CA, cerebellar ataxia; GI, Gait instability*.

The nature of the mutation might affect the activity of the EIF2B complex. Still, the genotype-phenotype correlation is not constant among cases (17). In 12 cases of R113H homozygotes, the mood/cognitive change, seizures, and other clinical features are different among the reported cases. Among all 21 patients, 57.3% (12/21) had cognitive decline or mood change, and different kinds of seizures or epilepsy were found in 52.4% (11/21).

Raini et al. (18) demonstrated the critical role of EIF2B in the coordination of the expression of nuclear and mitochondrial genes, with a negative effect of EIF2B partial loss-of-function on the coordination of cytoplasmic and mitochondrial translation programs, and highlighted the importance of mitochondrial function in VWM pathology. Keefe et al. (19) revealed a similar role for the EIF2B complex in zebrafish and humans, including impaired somatic growth, early lethality, effects on myelination, loss of oligodendrocyte precursor cells, increased apoptosis in the CNS, and impaired motor behavior.

Fogli et al. (20, 21) reported that many but not all the patients with ovarioleukodystrophy have mutations in genes encoding the subunits of EIF2B. The present study compiled the cases of ovarioleukodystrophy caused by *EIF2B5* gene mutations, but certain cases were caused by other gene mutations (22–27). Therefore, ovarioleukodystrophy caused by *EIF2B5* gene mutations should be a variant phenomenon of LVWM. The study of other mutations and other genes could ultimately explain the variability in the clinical manifestations among patients.

**Figure 3:**
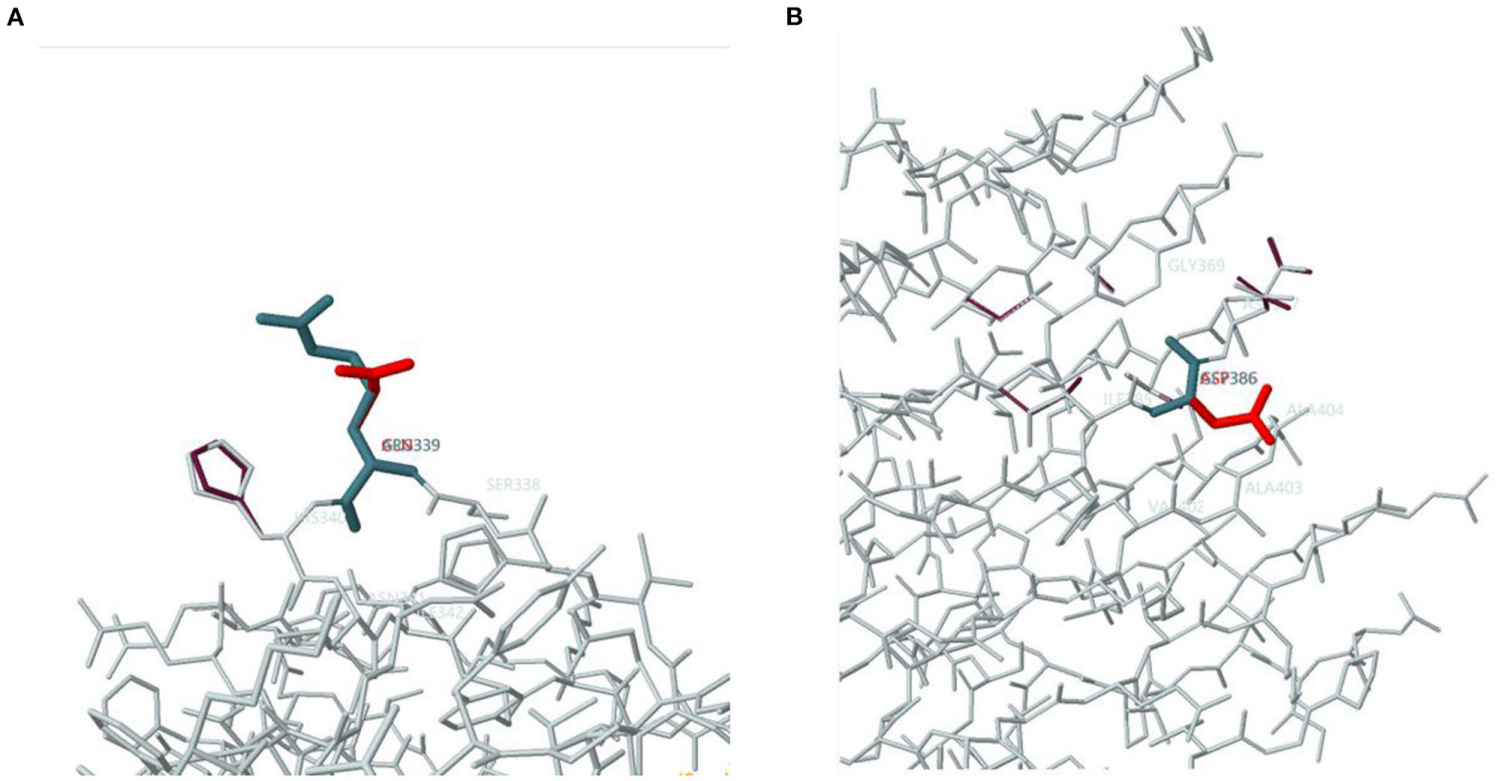
Simulated protein 3D structure based on the Phyre2 software (http://www.sbg.bio.ic.ac.uk/phyre2). Gray letters indicate the proper amino acids and red letters indicate abnormal amino acids encoded by gene mutations. The below mutation causes loss of Guanine nucleotide-exchange factor (GEF) activity in the Eukaryotic translation initiation factor 2B (elF2B) encoded by the EIF2B5 gene, which is unable to continuously catalyze the recycling of elF2 in peptide chain translation, thereby affecting the conversion of elF2- Guanosine diphosphate (GDP) into active eIF2- Guanosine triphosphate (GTP). **(A)** c.1016G>A (p.R339Q) in EIF2B5, position:339 in Protein Data Bank, Variation: Arginine (ARG) > Glutamine (GLN). **(B)** c.1157G>A (p.G386D) in EIF2B5, position:386 in Protein Data Bank, Variation: Glycine (GLY) > Aspartic Acid (ASP).

Clinical epigenetics animal research showed that homozygous EF2B5 (Ile98Met) I98M mutant mice exhibited abnormal gait, male and female infertility, and epileptic seizures (28). These symptoms are very similar to the characteristics of the clinical cases we summarized. A correlation analysis by Fogli et al. (20) revealed a correlation between the age at onset of the neurological deterioration and the severity of ovarian failure. Unfortunately, there is no more solid basic evidence to prove how the same mutation spot can simultaneously cause changes in brain white matter and ovarian failure. Computer simulation of three-dimensional protein structure can help understand the abnormal protein structure and pathogenicity caused by a gene mutation, estimate the abnormal location of subunits, functional domains, and biochemical activity. Slynko et al. (29) found that mutations that lead to severe disease mostly affect amino acids with pivotal roles in the complex formation and function of EIF2B. About 60% of mutations affect the ε-subunit, containing the catalytic domain, resulting in severe effects. About 55% affect subunit cores, with variable clinical severity. About 36% affect subunit interfaces, mostly with severe effects. Figure 3 shows the two abnormal protein 3D structures due to the ElF2B5 gene mutations observed in the patient reported here. According to the protein function prediction software (http://www.sbg.bio.ic.ac.uk/phyre2) (30), these two abnormal protein structures are highly likely to cause the disease. Unfortunately, there are no similar representations in the patient's family members, so we cannot obtain additional genetic verification. We hope that more studies will help to reveal this pathogenesis soon.

## Limitations

Adult-onset *EIF2B5*-related conditions are probably more frequent than initially thought, particularly in females (31). LVWM with ovarian failure was described in 1997 (10). Children or younger CACH/VWM patients with “ovarian dysgenesis” or “bilateral streak ovaries” (10, 21, 32–35), early LVWM with ovarian failure without genetic testing information 10, EIF2B5 gene variants related adult late-onset LVWM sporadic or family studies without ovarian failure evidence (2, 11, 36, 37), were not included in the present literature review. It is a strength of the present study, i.e., the included patients all share the same condition, but it is also a limitation since it prevents the study of all clinical manifestations of EIF2B5 mutations. Other not EIF2B5 gene variants ovarioleukodystrophy cases (22–27) are not included either. In addition, the symptoms of ovarian failure in women are easily missed, we did early as well. Due to some single case report or case-series articles of ovarioleukodystrophy lack precise information or data on ovarian failure, we have them out of the Table 1. Of course, the cases were obtained from the literature, and the analysis suffers from all the limitations of the included studies.

## Conclusion

In conclusion, a new genetic locus for LVWM was discovered in one patient. Compared with previous similar cases, different site mutations might have different clinical manifestations and obvious clinical heterogeneity.

## Data Availability Statement

The datasets presented in this study can be found in online repositories. The names of the repository/repositories and accession number(s) can be found in the article/supplementary material.

## Ethics Statement

The studies involving human participants were reviewed and approved by Shenzhen Traditional Chinese Medicine Hospital Ethics Committee. The patients/participants provided their written informed consent to participate in this study. Written informed consent was obtained from the individual(s) for the publication of any potentially identifiable images or data included in this article.

## Author Contributions

FK was a major contributor in designing the case report and drafting the manuscript. JW and ZG revised the manuscript. FK, XL, HZ, and SL gave the clinical information containing medical history, neurological findings, hematological examination, electrophysiological analysis, neuroimages evaluation, and treatment. All authors commented on previous versions of the manuscript, read, and approved the final manuscript.

## Funding

This work was supported by the Shenzhen Municipal Commission of Health and Family Planning [grant number SZFZ2018013]; National Natural Science Foundation of China [grant number 82004284]; Guangdong Medical Science Foundation [grant number A2020370]; Guangdong Administration of Traditional Chinese Medicine Project [grant numbers 20201419, 20211328, and 20221357]; Shenzhen Science and Technology Research Program [grant number RCBS20200714114959156].

## Conflict of Interest

The authors declare that the research was conducted in the absence of any commercial or financial relationships that could be construed as a potential conflict of interest.

## Publisher's Note

All claims expressed in this article are solely those of the authors and do not necessarily represent those of their affiliated organizations, or those of the publisher, the editors and the reviewers. Any product that may be evaluated in this article, or claim that may be made by its manufacturer, is not guaranteed or endorsed by the publisher.
